# The EnergyKids Pilot Study: Comparing Energy Balance of Primary School Children during School and Summer Camp

**DOI:** 10.3390/nu13010092

**Published:** 2020-12-30

**Authors:** Cinzia Franchini, Alice Rosi, Cristian Ricci, Francesca Scazzina

**Affiliations:** 1 Department of Food and Drugs, University of Parma, 43125 Parma, Italy; cinzia.franchini@unipr.it (C.F.); francesca.scazzina@unipr.it (F.S.); 2 Pediatric Epidemiology, Department of Pediatrics, Medical Faculty, Leipzig University, 04103 Leipzig, Germany; Cristian.Ricci@medizin.uni-leipzig.de; 3 Giocampus Scientific Committee, 43124 Parma, Italy

**Keywords:** energy balance, energy intake, energy expenditure, primary school, summer camp, physical activity, BMI, food record, activity tracker, children

## Abstract

Children’s energy requirements may vary during school and summer camp days. To evaluate energy balance during these two periods, seventy-eight children (45% females, 8–10 years) living in Parma, Italy, were enrolled in this observational study. Participants completed a 3-day food diary and wore an activity tracker for three consecutive days during a school- and a summer camp-week to estimate energy intake (EI) and energy expenditure (TEE). Height and body weight were measured at the beginning of each period to define children’s weight status. BMI and EI (school: 1692 ± 265 kcal/day; summer camp: 1738 ± 262 kcal/day) were similar during both periods. Both physical activity and TEE (summer camp: 1948 ± 312; school: 1704 ± 263 kcal/day) were higher during summer camp compared to school time. Therefore, energy balance was more negative during summer camp (−209 ± 366 kcal/day) compared to school time (−12 ± 331 kcal/day). Similar results were observed when males and females were analyzed separately but, comparing the sexes, males had a higher TEE and a more negative energy balance than females, during both periods. The results strongly suggest that an accurate evaluation of children’s energy balance, that considers both diet and physical activity, is needed when planning adequate diets for different situations.

## 1. Introduction

The ages corresponding to a child’s growth and development are a period of life characterized by an increase of organ size and the development of new functions; these changes entail the synthesis and deposition of new tissue, and, as a consequence, an increase of energy requirements [1]. Therefore, it is essential to always have an adequate energy intake (EI) to cover the organism’s needs, maintain physiological homeostasis, and ensure proper growth during childhood [2]. The daily energy requirement is based on the total energy expenditure (TEE) and is defined as the number of calories necessary to satisfy the TEE in a healthy and active organism [3]. The equilibrium between intake and expenditure of energy is called energy balance and is controlled by a neuroendocrine feedback mechanism, which not only regulates the food intake based on the body’s energy reserves but also maintains body weight over time [2,3]. Energy balance is reached when EI is equal to TEE, and during childhood, the energy cost of growth should be added to the energy spent [1]. Maintaining energy balance is essential to allow for adequate growth and for the full expression of a child’s biological and social potential. On the contrary, positive and negative energy balance conditions, if prolonged, are related to obesity or undernourishment and weight loss, respectively [4].

In Italy, as in other countries of the world, the incidence of overweight and obesity is increasing in all age groups, and in children it represents a high-risk for future health conditions [5]. In this context, creating a non-obesogenic environment by establishing healthy routine is essential in childhood obesity prevention. Improvements in children’s lifestyle by counteracting unhealthy behaviors as poor quality of diet, lack of physical activity, excessive sedentary/screen time, and inadequate sleep duration, have been proved to also improve the weight status [6,7,8,9]. The creation of a structured daily routine is very important for child development, because it promotes favorable activities and simultaneously reduces the risk of filling hours with sedentary activities [10]. In this framework, schools represent structured environments where the days are characterized by beneficial events such as physical activity, planned caloric intake and regular meal/snack times [11]. Furthermore, during school days children go to bed earlier and wake up earlier and several studies have highlighted that this is a correct habit for a child’s weight status [12,13]. On the contrary, during the summer holidays, children have usually more freedom to spend their time in unhealthy and sedentary activities, e.g., watching television and playing video games [14,15,16]. To overcome this problem, summer camps represent structured environments with organized leisure time activities, with a positive impact on children well-being. Besides the regular school schedule, participating in organized leisure time activities improves children’s development by positively affecting both physical and mental health [17,18]. Indeed, children’s physical activity level is influenced by the environment in which they live and a structured environment, like a summer camp, might reduce sedentary habits and promote physical activity [19,20]. In addition, children regularly attending a summer camp have healthier eating behaviors, demonstrating that summer camps could also provide benefits on children’s diets [19].

Although the positive impact of structured physical activity during children’s leisure time has been widely investigated in the past years, to the best of our knowledge, no study has assessed energy balance of primary school children by comparing their dietary intake and physical activity during school, a formal education setting, and summer camp, a non-formal education setting,. Apart from better lifestyle models adopted by children enrolled in summer camps during the holidays, it is important to consider that an increased physical activity level may result in higher nutritional needs to cover the cost of growth. Therefore, the aim of this study was to evaluate the energy balance (estimated as the difference between daily EI and daily TEE) during school days and summer camp days of primary school children living in the city of Parma (North Italy).

## 2. Materials and Methods

### 2.1. Study Design and Participants

**Figure 1:**
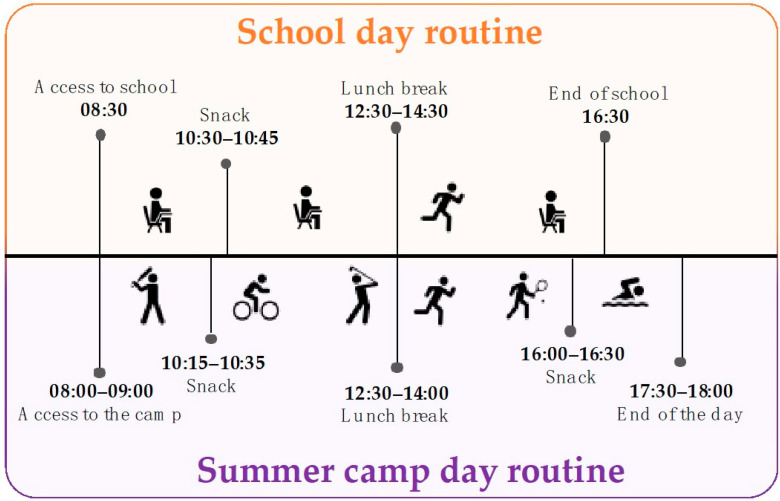
School day and summer camp day routine.

The EnergyKids project was an observational study, carried out during 2018 and 2019 in the city of Parma (North Italy) within the Giocampus Project [21,22]. Every year, the Giocampus project involves over 11,000 children aged 5 to 14 years during two different periods: school and summer. The school phase takes place from September to June, while the summer phase takes place from the end of the school year in June until the beginning of the new school year in September, in the form of a summer camp. Despite the same educational message based on a healthy lifestyle being pursued with continuity during both phases, the school setting limits physical activity to two hours per week of physical education. The school day routine is characterized by many hours of frontal lessons allowing very little physical activity during morning and lunch breaks. On the contrary, during the summer camp, children perform up to 6 h per day of physical activity doing both sports and active leisure occupations [18]. Children’s daily routines during a typical school day and summer camp day are shown in Figure 1.

When enrolling on the Giocampus summer camp, primary school children, 8–10 years old, were invited to participate in the study. The participation into the study was voluntary. Only children registered at the summer camp during the month of June or the month of September were eligibility for this study. Exclusion criteria were: metabolic syndromes, following chronic drug therapy for any pathology (including psychiatric disorders), following dietary therapy, and previously diagnosed food intolerance or food allergies. Study procedures were discussed with the children and their parents/legal tutors before obtaining a signed informed consent form.

The study was performed in compliance with the Helsinki declaration and the ethical approval was granted by the Ethical Committee of the Area Vasta Emilia Nord (Prot. n. 24169/2018).

Data were collected during a school week and a summer camp week, less than 4 weeks apart. Study design and time frame between the two data collections were chosen so as to reduce possible bias related to child growth or climate that may affect children’s body weight, height, and appetite. Depending on the week of participation at summer camp, children were divided into two subgroups. The first group was monitored at school first and later at the summer camp (in June), whilst the second group was monitored at the summer camp first and then at school (in September).

### 2.2. Personal Data and Anthropometric Measurements

For each subject, personal data (e.g., sex and age) were noted while anthropometric measurements (height and body weight) were taken, using the WHO guidelines [23], by qualified personnel in both assessment weeks. A portable stadiometer (Leicester Tanita HR 001, Tanita, IL, USA) and an electronic scale (MQ919, Maniquick, Niederkassel, Germany) were used to measure height to the nearest 1 mm and the body weight to the nearest 100 g respectively. During measuring, children wore a t-shirt and shorts and the body weight was later corrected using the Italian national surveillance system *Okkio alla Salute* method [24]. BMI was calculated as weight in kilograms divided by the square of the height in meters, and the weight status was evaluated through the IOTF gender- and age-related cut-offs for children BMI [25].

### 2.3. Energy and Nutrient Intake

Children’s food intakes were assessed through two semi-weighed 3-day food diaries, adapted for children, collected during three consecutive weekdays during a school week and for another three consecutive weekdays during a summer camp week. Children and parents/legal tutors were taught to fill in the diaries by recording all food and beverages consumed during the day. Teachers helped the children in registering the data of lunch and snacks consumed at school while qualified personnel helped to record food served for lunch and snacks at summer camp. Daily average values for energy, proteins, lipids, carbohydrates, and fiber were estimated through the Italian Food Composition Database for Epidemiological Studies in Italy of the European Institute of Oncology [26] as mean values of the three assessment days for each participant.

### 2.4. Total Energy Expenditure

The TEE of children was estimated by multiplying the Basal Metabolic Rate (BMR), obtained through Schofield’s predictive equation [27], by the physical activity level (PAL), the latter being calculated by means of an activity tracker (Fitbit Flex 2™, FitBit Inc., San Diego, CA, USA). Children wore the activity tracker during the school and the summer camp periods for the same three assessment days in which participants completed the food record. The activity tracker was delivered to the children on the afternoon before the beginning of the monitoring period and was to be worn before going to bed, and not be removed until the morning after the end of the assessment days. The activity tracker was used to record the physical activity data of each child by evaluating the minutes of inactivity and of light, moderate, and vigorous activity. Average data were calculated as mean values of the three assessment days for each subject.

### 2.5. Statistical Analysis

Sample representativeness was based on the number of primary school students aged 8 to 10 years living in Parma during the study period, which was around 2500 children. Keeping 90% level of confidence and 10% marginal error, the study population should be composed of at least 67 children to be representative of the whole population of 8–10 year old children living in Parma. As some participants could dropout during the study or could be excluded from the analyses due to missing values, around a hundred children were invited to participate in the study.

Data referred to continuous variables were expressed as mean ± standard deviation (SD) whereas categorical variables as absolute frequency or percentages. The normality of distribution was assessed through the Kolmogorov-Smirnov test. Descriptive statistics were performed to describe participants’ percentile of growth, daily energy and macronutrient intakes, physical activity, BMR, PAL, TEE, and energy balance. For all variables considered, differences between the school period and the summer camp period were investigated for the total sample and also separately for each sex by using a Student’s *t*-test for paired samples. Differences between sexes were also investigated by using a Student’s *t*-test for independent samples. The Pearson Chi-square test was used to evaluate associations between categorical variables (BMI categories × periods). Compositional data (time spent in physical activities of different intensity and nutrient intakes) were analyzed using the Isometric Log Ratio Transformation (ILR). Briefly, a complex of D-dimensional compositional data (whose sum would result in a constant value) was transformed to a D-1 dimensional Euclidean vector [28]. Ternary plots were used to represent compositional data. Mixed model analyses were performed using a random slope and random intercept models selected over simpler models using the Bayesian Information Criteria (BIC). The association between covariates and TEE were reported by means of effect size, t values and *p* values for null effects. The IBM SPSS Statistics for Macintosh, version 25.0. (IBM Corp., Armonk, NY, USA and R software version 3.6 were used to perform the statistical analyses, type-I error rate was set to 5% (α = 0.05). The ilr function (compositions package) and lme (nlme package) function were used to perform the Isometric Log Ratio Transformation and mixed model, respectively.

## 3. Results

Out of a total of 105 enrolled children, four did not complete the study and 23 were excluded for missing/under-reported data. Thus, a total of 78 participants, 44% females and 56% males, with a mean age of 9 ± 1 correctly completed all study requests. Anthropometric and behavioral data of the total sample are shown for both assessment periods in Table 1.

No differences were found for height and BMI, while body weight was significantly higher during school days (*p* = 0.027). The mean BMI corresponded to a normal weight status in both periods, and more than 70% of children was normal weight. Moreover, the children’s distribution among the BMI categories was similar between the two assessment periods and no significant association was found between weight status and attending school or summer camp (X^2^ = 0.664; df: 3; *p* = 0.882). Regarding dietary intakes, no significant differences were found between the two periods for energy and carbohydrate intake, whereas protein, lipid, and dietary fiber intakes were higher during the summer camp period (*p* = 0.003; *p* = 0.030; *p* = 0.002; respectively). Significant differences were found for the time spent in inactivity/sleep or doing light, moderate, or vigorous activities. Compared to school days, during the summer camp period the minutes of inactivity/sleep were lower (*p* < 0.001), whereas the minutes of light, moderate and vigorous activities were higher (*p* < 0.001, for all). Consequently, during summer camp significantly higher PAL (*p* < 0.001) and TEE (*p* < 0.001) were found compared to the school period. Lastly, children’s energy balance was found to be negative in both assessment periods, but during the summer camp days it was significantly more negative than during the school days (*p* < 0.001).

Differences between girls and boys were studied in both periods and participants’ characteristics are shown by gender for each assessment period in Table 2. In both sexes, the mean BMI corresponded to a normal weight status and no significant differences were found between males and females, however females had a marginally higher weight (school: *p* = 0.046; summer camp: *p* = 0.029) and were taller than males in both assessment periods (school: *p* = 0.033; summer camp: *p* = 0.030). Energy and nutrient intakes were similar between boys and girls in both assessment periods. Significant differences were observed for the time spent in light (school: *p* = 0.043; summer camp: *p* = < 0.001), moderate and vigorous (*p* = < 0.001, for both parameters and periods) activities, and inactivity/sleep time during summer camp days (*p* = 0.004), highlighting the fact that males were more physically active than females in both periods. BMR was similar between girls and boys during both school and summer camp days. On the contrary, the PAL and TEE were higher in males than in females (*p* < 0.001, for both periods). The energy balance was more negative for boys than for girls in both school (*p* = 0.023) and summer camp (*p* = 0.006) days.

**Figure 2:**
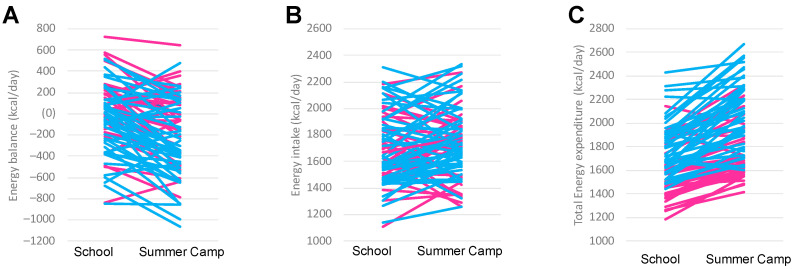
Energy balance (**A**), energy intake (**B**), and total energy expenditure (**C**) of individual participants during school days and summer camp days. Pink lines are females, whereas blue lines are males.

Differences between school and summer camp for girls and boys were also separately analyzed (Table 2). When only female participants were considered, body weight, height, BMI and BMR, as well as EI were found to be similar between periods. The time spent in light, moderate, and vigorous physical activities, PAL, TEE, and energy balance were all significantly higher during summer camp days (*p* < 0.001, for all). The same results were found for male participants, except for body weight that was higher during school days compared to summer camp days (*p* = 0.024). A graphical representation of individual EI, TEE and energy balance for all participants on school days and summer camp days is showed in Figure 2.

A negative slope of energy balance between school days and summer camp days was observed for the majority of the sample (74%), being more negative during the summer camp period, both for males (77%) and females (71%). Considering the difference between EI during the two assessment phases, around half of the sample (58%) reported a higher intake during the summer camp days, 47% and 66% of females and males, respectively. On the contrary, TEE was higher during the summer camp days in the majority of children (91% for total samples, for males, and for females).

**Figure 3:**
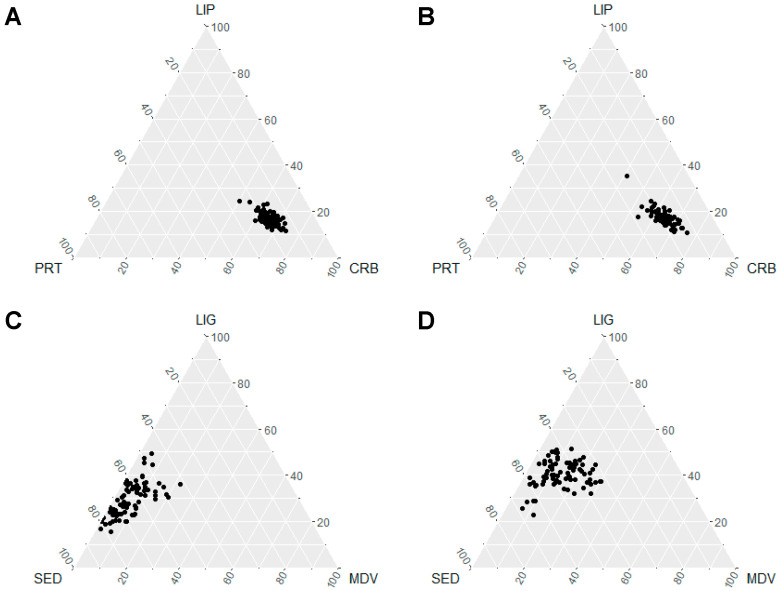
Ternary plot for dietary composite data during school days (**A**) and summer camp days (**B**), and for physical activity composite data during school days (**C**) and summer camp days (**D**). CRB: carbohydrates; LIP: lipids; PRT: proteins. LIG: light activities; MDV: moderate-vigorous activities; SED: sedentary activities (inactivity/sleep).

Compositional data referred to EI and TEE components during the two assessment phases are showed in ternary diagrams (Figure 3), considering the relative contribution of carbohydrates, lipids and proteins, and the time spent in sedentary, light, and moderate-vigorous activities, respectively.

Finally, the mixed model analyses confirmed the above results with higher TEE in males compared with females when considering models with random intercept and random slope for summer camp participation (Table 3). On the other hand, TEE was consistently higher during the period of summer camp participation with a difference of about 240 kcal/day. A similar association was observed also for the ILR of moderate and vigorous physical activity. Finally, the ILR of nutrients intake was not associated to TEE in any of the model considered.

## 4. Discussion

This pilot study gives new information on the energy balance during school days and summer camp days in a sample of healthy primary school children (aged 8–10 years) living in Parma (North Italy). Even if physical activity and TEE during the summer camp days were expected to be higher than during school days, the EI could have been different or remain the same during the two periods, covering or not the energy needs. Our results highlighted that daily EI and TEE were approximately balanced during school days whereas energy balance was negative when children attended the summer camp.

A detailed comparison of our results with other studies is difficult because only few studies have explored children energy balance calculating EI and TEE during the same assessment days. EI, estimated using a 7-day food record, and TEE, derived from accelerometers, of 81 Danish schoolchildren aged 8–11 years, were found to be almost balanced during the school term (mean difference: −4 kcal/day) [29]. On the contrary, a negative energy balance (around −300 kcal/day) was reported for 8–9 years Norwegian children when, in line with our results, energy balance was more negative for boys than for girls [30]. However, some limitations in the assessment methods of the Norwegian study may have biased this result, as the use of a web-based food recall may have increased the number of under-reporter children. To the best of our knowledge, no study has explored differences in energy balance between school and summer camp days. In our study, TEE was found to be higher than EI when children were at the summer camp. This unbalanced situation was due to a higher expenditure linked to an increased physical activity level not counterbalanced by a higher EI. About this, food intake and altering TEE due to physical activity changes can be defined as “energy balance-related behaviors” (EBRBs) [31]. The relationship between these two EBRBs is not linear but influenced by environmental and lifestyle factors [32]. Indeed, the TEE does not necessarily drive-up food intake to balance energy needs, even when the increase in physical activity should be large enough to stimulate appetite. In this complex regulation mechanism, the intensity, duration, and kind of activity influence food intake. But it should be kept in mind that sedentary behaviors can also stimulate the appetite, overcoming real body’s signals of hunger and satiety [33]. These are very crucial aspects considering that children energy needs are essential to ensure a proper growth and mental development.

EBRSs are generally responsible for weight changes. In this study, body weight was significantly higher during school days. The lower body weight measured during the summer camp, especially for male participants who also had a more negative energy balance, might be linked to the negative energy balance calculated during this assessment phase. Regarding weight status, the majority of children was classified as normal weight (72–73%). Only one participant (1%) was obese, in contrast with local (5–7%) [34,35,36,37,38,39,40], regional (8%) and national data (9%) [33]. Likewise, the percentage of overweight children (school days: 15%; summer camp days: 13%) was similar to that observed in a similar sample of children living in the same city (15%) [26] but lower than other local (23%) [32], regional (21%), and national data (21%) documented by the last national surveillance system [41].

The EI was marginally lower than the amount recommended for 8–10 year-old Italian children that should be between 1700 and 2500 kcal on the basis of children’s age and PAL [42]. During school days, when the PAL was lower and children spent several hours in sedentary activities, the EI registered was almost adequate to cover the TEE, while during summer camp days, when children spent the day playing outside and doing sports, it was not sufficient to cover their energy needs.

The EI reported by children in the present study was slightly higher of the one described in another study of similar-aged children, living in the same city, who used the same 3-day food diary during the school period [34], and also of the EI reported in a second study on 7–11 year-old children living in Northern Italy in which a similar 3-day food record was used to collect dietary data [35]. These data may exclude the possibility that the negative energy balance observed in this study could be due to under-reported intakes. Higher intakes, around 2000 kcal/day, have been reported for pre-school and school-age Italian children [28,29] but differences in the assessment methods could make it difficult to compare data. Irrespective of energy, in both assessment periods, the children’s diet was nutritionally balanced considering total EI and in line with National [42] and international guidelines [38,39] for carbohydrates, lipids, and dietary fibers. However even if protein intake was only slightly higher than the 10% of EI recommended for 8–10 year-old males and females, it was within the12–18% range of energy, considered a reasonable intake [38,39,42].

When children were grouped by sex, only females had an EI adequate enough to cover their TEE and to satisfy their energy requirements, while males were unable to meet their energy requirements due to an insufficient EI to cover their needs during both school and summer camp days. This result concurs with another Italian study in which females had an EI closer to their estimated TEE when compared to males [36].

The WHO recommends at least 60 min of moderate-to-vigorous physical activity (MVPA) every day and activities that strengthen muscles and bones at least 3 times a week for children and adolescents aged 5–17 years [43]. In our study, participants showed a physical activity level in line or even higher than the WHO recommendations. As expected, children were less active during school days, and in both periods, girls performed on average less physical activity than boys (MVPA: 48 min/day vs. 98 min/day during school, and 86 min/day vs. 168 min/day during summer camp days). So, during summer camp the WHO recommendations were met and exceeded by all children, however during the school period only males met the activity goals whereas females were slightly below the goals. These results confirmed gender differences highlighted by a recent European study in which 419 11-year-old children from Germany, Belgium, Italy, Poland, and Spain were involved [44]. This recent work showed males spending more time on high-intensity activities than females, who instead spent more time on low-intensity activities. Moreover, in line with our results, seasonal differences were found, and the time spent on MVPA resulted lower during winter compared to summer [44]. In addition to this study, gender differences were confirmed by Pérez-Rodrigo and colleagues, whose findings also demonstrated that Spanish children with a healthier diet spent more time in MVPA and walking time compared to their peers with a poorer diet, thus highlighting the importance of healthy lifestyle patterns which include both a proper diet and physical activity [45]. Furthermore, recent European studies have shown that the time spent in MVPA decreases with age while sedentary behaviors increase [46,47]. These results suggest a key role of the “Giocampus Estate” summer camp in the promotion of an adequate physical activity level among school-age children who may not be active enough during the school year.

Despite the fact that some positive results were observed, our study has some limits. Due to the small sample size and the limited enrollment area, the results are local and may not be generalized to all Italian primary school children; despite this, our study is relevant because it can be considered a starting point for future research. The low percentage of overweight-obese children is a very positive result. However, it should be considered that the participation to the study was voluntary and it may be possible that obese children did not attend the summer camp or did not adhere to the project. In addition, results were based on data collected during three consecutive days that may not be representative of the actual daily routine of children during both school and summer camp days. Monitoring children habits and anthropometric variables for longer periods in both settings would be very interesting and should be further considered. Regarding the food intake assessment, the food diary is the most accurate method, but it requires a high-level of commitment, which is difficult for a child, so an adult’s help is fundamental. Due to this fact four participants dropped out of the study and some participants were excluded from the analyses due to missing or under-reported data. Despite this, the food diary is a strength of this study because it gives an accurate estimate of the actual food intake. Another asset is the use of an activity tracker to evaluate the PAL and sleep duration as it is a useful non-invasive device for evaluating the level of physical activity. In fact, the Fitbit Flex 2™ has been proven to be accurate in recording physical activity, representing a suitable alternative to accelerometer, in particular in children [48]. Moreover, the Fitbit Flex 2™ used in this study does not have a display so children could not see their daily results or be conditioned by it during their daily routine.

## 5. Conclusions

To the best of our knowledge, this was the first study that analyzed children’s energy balance, in both a school setting and a summer camp, with the aim of assessing whether the dietary intake of children was adequate to cover their needs during both school and summer camp days.

Due to the small sample size, this study is just a pilot study. However, our results highlight the importance of not only providing children with nutritionally adequate lunches and snacks but also of accurately evaluating children’s energy balance for planning adequate diets in different settings by considering both their dietary needs and physical activity. More detailed dietary guidelines are strongly recommended to help and guide food caterers of schools and summer camps in developing customized menus on the basis of the real nutritional needs of children. Our findings should not be overlooked because a protracted negative energy balance could cause undernourishment, and consequently, poor growth during development while, on the other hand, a prolonged positive energy balance could lead to overweight and obesity. Further investigations are needed to explore this important aspect related to children’s proper growth.

## Figures and Tables

**Table 1:** Anthropometric measurements and behavioral data for the total sample during the two assessment periods.

Variable	School Days (*n* = 78)	Summer Camp Days (*n* = 78)	*p* Value *
Weight (kg)	31.3 ± 7.0	31.2 ± 6.8	0.027
Height (cm)	134.9 ± 7.5	134.8 ± 7.5	0.108
BMI (kg/cm^2^)	17.0 ± 2.3	17.0 ± 2.3	0.145
BMI category (n (%))			0.882 ^§^
Underweight	8 (10)	11 (14)	
Normal weight	57 (73)	56 (72)	
Overweight	12 (15)	10 (13)	
Obese	1 (1)	1 (1)	
Energy intake (kcal)	1692.4 ± 265.2	1738.3 ± 261.6	0.084
Proteins (g)	63.9 ± 12.4	68.0 ± 12.4	0.002
Energy (%)	15	16	
Lipids (g)	59.0 ± 13.2	62.3 ± 13.4	0.030
Energy (%)	31	32	
Carbohydrates (g)	233.0 ± 39.5	232.9 ± 44.1	0.983
Energy (%)	55	54	
Dietary fibers (g)	15.8 ± 4.7	17.7 ± 5.1	0.002
Energy (%)	2	2	
Inactivity/Sleep (min)	1039.7 ± 64.6	923.7 ± 59.0	<0.001
Light activity (min)	324.2 ± 53.2	384.9 ± 48.6	<0.001
Moderate activity (min)	45.1 ± 24.0	78.0 ± 33.8	<0.001
Vigorous activity (min)	31.0 ± 25.4	53.4 ± 33.4	<0.001
Basal Metabolism Rate (kcal)	1163.4 ± 129.9	1159.3 ± 125.7	0.028
Physical Activity Level	1.5 ± 0.2	1.7 ± 0.2	<0.001
Total Energy Expenditure (kcal)	1704.0 ± 262.3	1947.5 ± 312.3	<0.001
Energy balance (kcal)	−11.6 ± 331.4	−209.2 ± 366.4	<0.001

Data are presented as mean ± SD for continuous variables and frequency (% of total sample) for BMI category and as percentage for the macronutrient contribution to the energy intake. * Student’s *t*-test for paired samples. ^§^ Chi-square test.

**Table 2:** Anthropometric measurements and behavioral data by gender during the two assessment periods.

Variables	School Days			Summer Camp Days			*p* Value ^§^	
	Female (*n* = 34)	Male (*n* = 44)	*p* Value *	Female (*n* = 34)	Male (*n* = 44)	*p* Value *	Female	Male
Weight (kg)	33.2 ± 8.3	29.9 ± 5.4	0.046	33.2 ± 8.1	29.6 ± 5.3	0.029	0.520	0.024
Height (cm)	137.1 ± 8.8	133.3 ± 5.9	0.033	137.0 ± 8.8	133.1 ± 6.0	0.030	0.511	0.109
BMI (kg/cm^2^)	17.4 ± 2.7	16.7 ± 2.0	0.181	17.4 ± 2.7	16.6 ± 1.8	0.110	0.964	0.078
Energy intake (kcal)	1660.2 ± 250.4	1717.2 ± 276.3	0.350	1679.2 ± 252.8	1783.9 ± 261.9	0.079	0.652	0.053
Proteins (g)	61.1 ± 10.3	66.1 ± 13.5	0.077	65.9 ± 13.0	69.6 ± 11.9	0.187	0.024	0.044
Energy (%)	15	15		16	16			
Lipids (g)	57.8 ± 13.4	59.9 ± 13.2	0.484	61.4 ± 16.2	63.1 ± 10.9	0.575	0.168	0.094
Energy (%)	31	31		33	32			
Carbohydrates (g)	229.9 ± 40.5	235.3 ± 39.0	0.557	223.9 ± 40.4	239.8 ± 45.9	0.116	0.450	0.465
Energy (%)	55	55		53	54			
Dietary fibers (g)	16.1 ± 5.0	15.6 ± 4.5	0.605	16.8 ± 4.5	18.3 ± 5.5	0.186	0.444	0.001
Energy (%)	2	2		2	2	2		
Inactivity/Sleep (min)	1054.1 ± 75.5	1028.6 ± 52.9	0.098	945.4 ± 51.3	906.9 ± 59.6	0.004	<0.001	<0.001
Light activity (min)	338.0 ± 61.4	313.5 ± 43.8	0.043	409.9 ± 46.3	365.5 ± 41.4	<0.001	<0.001	<0.001
Moderate activity (min)	32.3 ± 18.3	54.9 ± 23.3	<0.001	55.0 ± 23.0	95.7 ± 30.0	<0.001	<0.001	<0.001
Vigorous activity (min)	15.5 ± 17.5	43.0 ± 24.2	<0.001	29.7 ± 15.0	71.8 ± 32.2	<0.001	<0.001	<0.001
Basal Metabolism Rate (kcal)	1143.6 ± 145.9	1178.7 ± 115.4	0.254	1142.3 ± 141.5	1172.5 ± 111.9	0.295	0.552	0.028
Physical Activity Level	1.4 ± 0.1	1.5 ± 0.1	<0.001	1.5 ± 0.1	1.9 ± 0.2	<0.001	<0.001	<0.001
Total Energy Expenditure (kcal)	1575.7 ± 243.8	1803.1 ± 233.4	<0.001	1759.8 ± 238.5	2092.4 ± 285.6	<0.001	<0.001	<0.001
Energy balance (kcal)	+84.5 ± 324.5	−85.9 ± 320.7	0.023	−80.6 ± 337.9	−308.5 ± 360.1	0.006	0.001	<0.001

Data are presented as mean ± SD for continuous variables and as percentage for the macronutrient contribution to the energy intake. * Independent-sample Student’s *t*-test for between sex analyses within each period. ^§^ Paired-sample Student’s *t*-test for between period analyses within females and males separately.

**Table 3:** Effect size differences of random slope for summer camp participation and random intercept mixed models evaluating TEE in relation to sex, participation to the summer camp, physical activity and nutrients intakes.

Model 1 -Sex and Camp Participation-				
Comparison	Effect size	Std.err	*t*-value	*p* Value
Males vs. females	280.04	53.76	5.21	<0.001
School vs. Camp	−243.47	21.36	−11.40	<0.001
**Model 2 -Sex, Camp Participation and Physical Activity-**				
Comparison	Effect size	Std.err	*t*-value	*p* Value
Males vs. females	113.91	65.18	1.75	0.0847
School vs. Camp	−243.47	21.36	−11.40	<0.001
ILR—Light physical activity	−171.47	104.92	−1.63	0.1065
ILR—Moderate + vigorous physical activity	222.44	53.19	4.18	<0.001
**Model 3 -Sex, Camp Participation and Nutrition Intakes-**				
Comparison	Effect size	Std.err	*t*-value	*p* Value
Males vs. females	273.06	54.99	4.97	<0.001
School vs. Camp	−243.47	21.36	−11.40	<0.001
ILR of Protein intake	132.53	176.30	0.75	0.4546
ILR of Lipids intake	−95.37	120.05	−0.79	0.4295
**Model 4 -Sex, Camp Participation, Physical Activity and Nutrition Intakes-**				
Comparison	Effect size	Std.err	*t*-value	*p* Value
Males vs. females	93.02	66.47	1.40	0.1660
School vs. Camp	−243.47	21.36	−11.40	<0.001
ILR—Light physical activity	−185.73	105.64	−1.76	0.0830
ILR—Moderate + vigorous physical activity	244.68	54.12	4.52	<0.001
ILR of Protein intake	99.55	163.20	0.61	0.5438
ILR of Lipids intake	−146.95	110.51	−1.33	0.1878

ILR: Isometric Log Ratio Transformation. Inactivity and carbohydrates were excluded from the D-part Aitchison-simplex isometrically to perform the D-1 dimensonal euclidian vector for physical activity and nutrition intake, respectively.
